# Effect of the Bacterial Chaperones SecB and Trigger Factor (TF) on the Folding Dynamics and In Vitro Translocation of Cytoplasmic and Secretory Model Proteins

**DOI:** 10.3390/ijms262311485

**Published:** 2025-11-27

**Authors:** Ying Xu, Haitham Sedky, Dries Smets, Jochem Smit, Spyridoula Karamanou, Anastassios Economou, Kurt Vermeire

**Affiliations:** 1 Department of Microbiology, Immunology and Transplantation, Rega Institute for Medical Research, KU Leuven, 3000 Leuven, Belgium; ying.xu1@kuleuven.be (Y.X.); haitham.abdallah@kuleuven.be (H.S.);; 2 Department of Microbiology and Immunology, Faculty of Pharmacy, Assiut University, Assiut 71526, Egypt

**Keywords:** protein secretion, protein folding dynamics, chaperones, trigger factor, SecB

## Abstract

Nascent polypeptides selected for export are synthesized in the cytoplasm by ribosomes and inserted into or translocated across membranes to reach their correct location. Exported proteins delay their folding and remain soluble during their cytoplasmic transit to the membrane. In bacteria, most secretory proteins require additional support from cytosolic chaperones such as trigger factor (TF) and SecB to promote their translocation competence. Here, we investigate the effect of TF and SecB on the folding dynamics and in vitro translocation of secretory and cytoplasmic model proteins PpiA and PpiB, respectively. Global hydrogen—deuterium exchange mass spectrometry (HDX-MS) experiments reveal that SecB delays the folding of slow-folding PpiA proteins but has no effect on fast folders like PpiB. In vitro protein translocation results show that TF inhibits the Sec-dependent translocation of mature PpiA/B and derivative proteins, as well as some secretory preproteins carrying a signal peptide (SP), whereas SecB has no clear effect under the same conditions. However, SecB proves to be dominant over TF in protein translocation in vitro. Finally, for the secretory preprotein proPpiA, SecB prevents SP-induced aggregation. Our findings indicate that the combined properties of signal peptides and mature domains dictate chaperone specificity and translocation efficiency, with both TF and SecB acting in a substrate-specific manner.

## 1. Introduction

Secretory polypeptides are synthesized in the cytoplasm and inserted into or cross the plasma membrane to reach their correct location [1,2]. In bacteria, one-third of the proteome is trafficked via the essential and ubiquitous Sec pathway [1]. In the model organism *E. coli*, nascent chains destined to be inserted into the inner membrane are mainly translocated co-translationally, whereas the remaining ~500 secretory proteins are translocated post-translationally after their synthesis is complete [1,3]. Mature domain Targeting Signals (MTSs) and N-terminal Signal Peptides (SPs) are recognized by the SecA subunit of the translocase, an essential ATPase, and activate the holoenzyme for translocation [4,5,6,7]. To cross SecYEG, preproteins need to avoid premature folding, misfolding or aggregation and remain non-folded and soluble [8]. Unique structural and sequence features of preproteins in both SPs and Mature Domain (MD) regions [4,8], together with chaperones [1,9] enable secretory clients to retain loosely folded states that are compatible with translocation. Despite the large number of cytoplasmic chaperones [9,10] only two have been directly implicated in protein secretion, i.e., SecB and trigger factor (TF) [11,12,13,14,15].

TF is a highly abundant chaperone [12], ubiquitous in bacteria. It forms dimers in the cytoplasm [16] and binds to ribosomes as a monomer that leans over the exit tunnel and scans nascent chains co-translationally [17,18]. TF interacts with ~20% of cytoplasmic proteins according to ribosome profiling [19] and acts as a quasi-foldase for a very small number (~5%) of aggregation-prone proteins in vitro [20]. In addition, TF may help sort secretory proteins as it recognizes both the SP and MDs [13]. Some of its secretory clients, such as proOmpA, are shared with other chaperones like SecB [13].

SecB is limited to proteobacteria and forms a tetramer, i.e., a dimer of dimers [21], that binds nascent preproteins post-translationally [22,23]. SecB specifically recognizes SPs and MD regions enriched in aromatic (Phe, Tyr, and Trp) and basic (Arg and Lys) residues [24,25], thought to be exposed when the protein is unfolded. In contrast to a generic recognition pattern, proteomics studies suggested that SecB may only recognize 25 preprotein clients (~5% of secretome) [26,27]; not all of them are SecB-dependent, but SecB may facilitate efficient trafficking. The most well-known and studied SecB client is proMBP [24,25].

We previously exploited two structural twins, the peptidyl-prolyl cis-trans isomerase A (PpiA; secreted; slow folder) and B (PpiB; cytoplasmic; fast folder), to define intrinsic features of preproteins that affect the folding rate [28]. These features were pinpointed with such accuracy that grafting a small number of residues between these nearly identical structures led to concomitant grafting of folding properties [28]. We additionally identified a dynamics crosstalk mechanism by which SPs delay early foldon formation, thus, MD folding [29].

In this study, we used the PpiA/B structural twins and derivatives to investigate whether chaperone interactions affect intrinsic folding dynamics of clients and, thus, govern the cytosolic sorting and secretion fate of proteins.

## 2. Results

### 2.1. SecB Delays Folding of Slow-Folding Proteins

**Figure 1:**
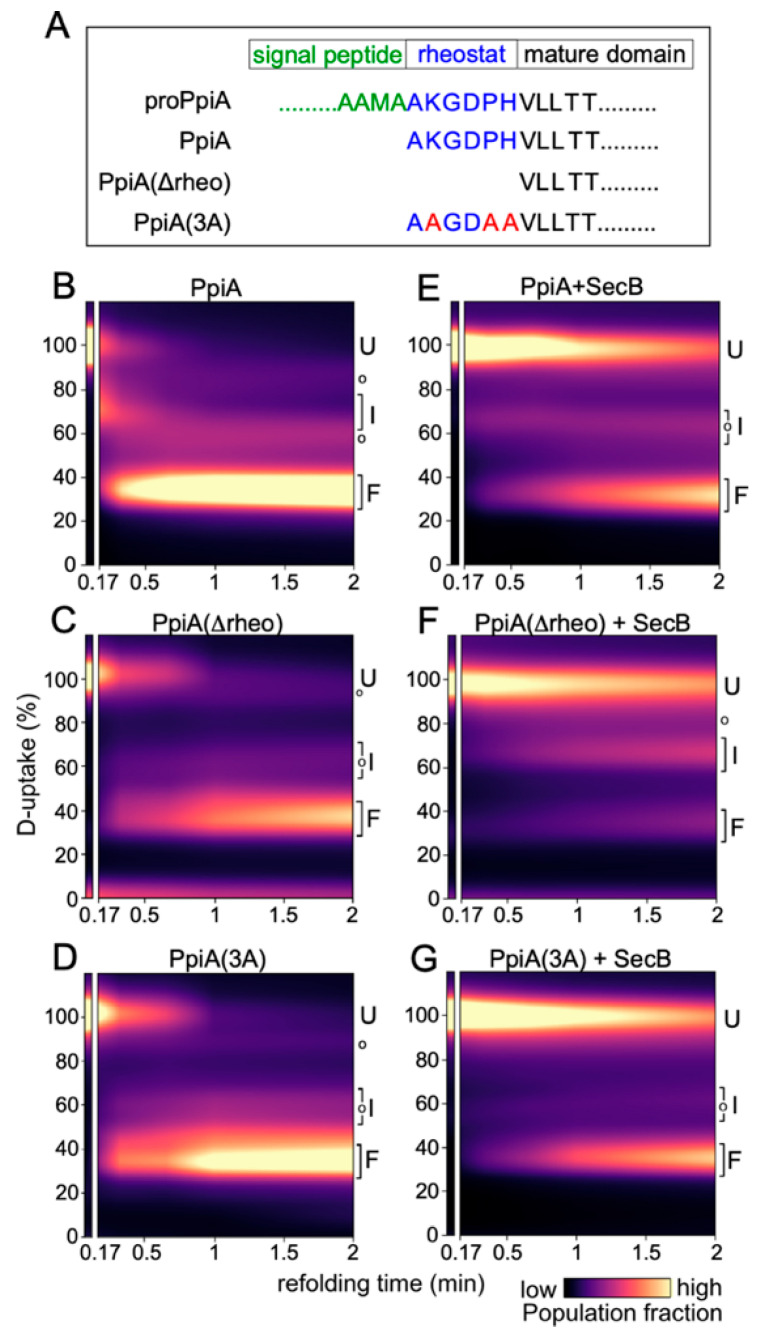
Sequence comparison of PpiA and its folding-altering variants and the effect of SecB on folding dynamics of these proteins. (**A**) Schematic alignment of the N-terminal sequences of proPpiA, PpiA, and their mutants used in this study. The signal peptide (green), rheostat region (blue), and mature domain (black) are indicated. Modified residues are shown in red. Dotted lines indicate omitted regions beyond the N-terminal portion. (**B**–**G**) The refolding of the indicated PpiA derivatives, at 25 °C, in the absence or presence of SecB (added at 1.5 molar excess over the Ppi proteins), was monitored by global Hydrogen–Deuterium Exchange Mass Spectrometry (HDX-MS) analysis. The Deuterium-uptake at each time point was expressed as a percentage of the D uptake of the fully unfolded state (6 M urea; set as 100%) and is shown as a continuous colour map of the population fraction over time. The Unfolded (U), Intermediate (I) and Folded (F) states, and modifications/adducts that are not part of the folding pathway (o) are indicated. *n* ≥ 2 biological repeats.

**Figure 2:**
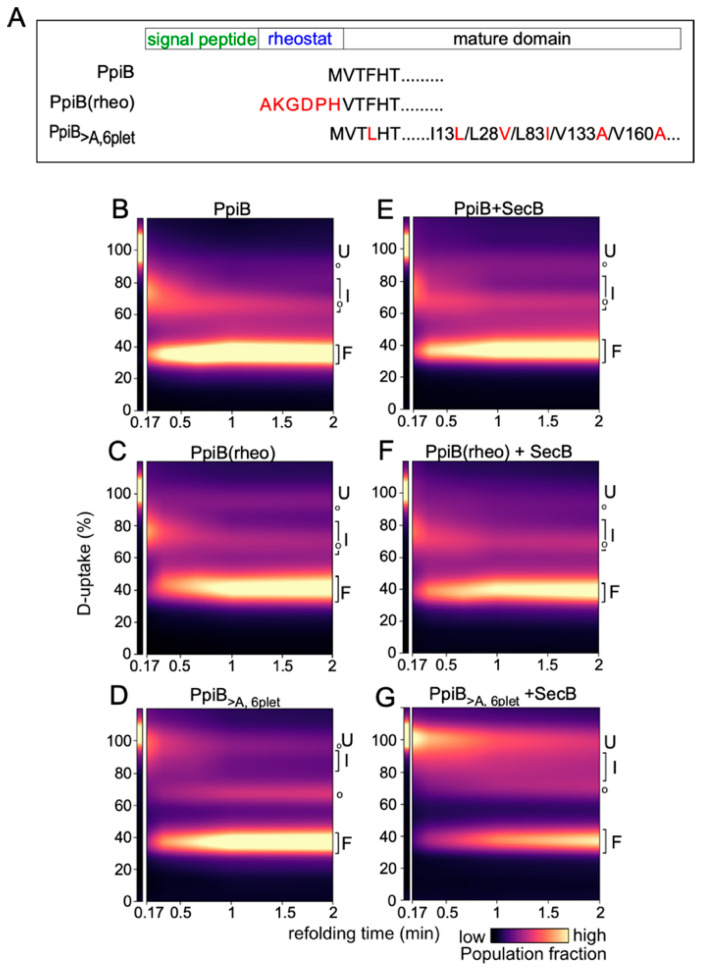
Sequence comparison of PpiB and its folding-altering variants and the effect of SecB on folding dynamics of these proteins. (**A**) Schematic alignment of the N-terminal sequences of PpiB and its mutants used in this study. The signal peptide (green), rheostat region (blue), and mature domain (black) are indicated. Added or modified residues are shown in red. Dotted lines indicate omitted regions beyond the N-terminal portion. (**B**–**G**) Refolding of the indicated PpiB derivatives, at 25 °C, in the absence or presence of SecB (added at 1.5 molar excess over the Ppi proteins), by HDX-MS. The Deuterium-uptake at each time point was expressed as a percentage of the D uptake of the fully unfolded state (6 M urea; set as 100%) and is shown as a continuous colour map of the population fraction over time. The Unfolded (U), Intermediate (I) and Folded (F) states, and modifications/adducts that are not part of the folding pathway (o) are indicated. *n* ≥ 2 biological repeats.

Previous work from our laboratory established that differences in the folding kinetics of the structural twins PpiA (periplasmic) and PpiB (cytoplasmic) significantly influence their capacity to be secreted via the Sec translocation pathway [28]. The signal peptide, the N-terminal early mature domain of PpiA (i.e., rheostat; Figure 1A), and native contacts in the mature protein all play important roles in protein folding and translocation [28,29]. In this study, we aim to investigate the role of SecB and TF chaperones in the folding kinetics of these structural twin proteins. In addition to the wild type (WT) protein, two mutants for each twin are included (Figure 1A and Figure 2A): PpiA(Δrheo), in which the six “rheostat” residues were deleted; PpiA(3A), in which three Ala residues were introduced in the rheostat (as indicated; Figure 1A); PpiB(rheo), in which the rheostat region of PpiA was added to PpiB (Figure 2A); and PpiB_>A,6plet_, in which six native contact residues from PpiA were introduced into PpiB.

To study the effect of chaperones on protein folding dynamics, we performed global hydrogen–deuterium exchange mass spectrometry (HDX-MS) experiments using purified proteins, as described previously [28,29]. In folded proteins amide hydrogens are less accessible for deuterium exchange as they are largely involved in hydrogen bonding or buried in the protein folding core. On the contrary, backbone amides are more solvent-accessible and exchangeable in flexible and/or unfolded proteins (with weak or no H-bonds). As a consequence, deuterium (D)-uptake inversely correlates with the extent of folding [30]: high D uptake indicates an unfolded state, low D uptake a more folded state.

Briefly, urea-denatured purified proteins were allowed to refold by dilution in native buffer I (18 µM protein; 0.2 M final urea concentration), in the absence or presence of a chaperone, at 25 °C. At various time points, protein aliquots were pulse-labelled in D_2_O, quenched, and analyzed on MS. Protein folding is seen as the progressive shift over time of one charged peak from the high m/z value of the unfolded state (U) towards the lower m/z value of the natively folded state (reflecting high-to-low D uptake). The degree of non-foldedness (D uptake) of the fully unfolded protein (U) is set as 100%; all other values are expressed relative to this one.

As shown in Figure 1, the refolding dynamics at 25 °C were monitored over a 2-min time course (x-axis) and expressed as population fractions of three visible structural states (y-axis): the unfolded (U; 100% D uptake), intermediate (I; 50–90% D uptake) and final folded (F; ~40% D uptake) states. Colour maps that were generated by Lorentzian fitting highlight population densities (Figure 1; purple = low; yellow = high). For instance, the slow folder PpiA (Figure 1B) remained in the unfolded state for ~20 s (top left area of colour map), transitioned to an intermediate, and then rapidly to the folded state (yellow area at bottom of colour map). The two PpiA mutants (Figure 1A) displayed delayed folding compared to their WT counterpart. In fact, PpiA(Δrheo) and PpiA(3A) both remained in the unfolded state longer than PpiA WT (Figure 1; compare C and D to B). Interestingly, the presence of SecB markedly delayed the folding of either WT PpiA (Figure 1; compare E to B) or the PpiA mutants PpiA(Δrheo) (compare F to C) and PpiA(3A) (compare G to D).

Unlike PpiA (Figure 1), the fast folder PpiB instantly entered the intermediate state while concurrently beginning to fold (Figure 2B). Mutant PpiB(rheo) exhibited a folding trajectory similar to WT PpiB, with a minor intermediate fraction before complete folding (Figure 2; compare C to B). Mutant PpiB_>A,6plet_ (i.e., the more PpiA “look-alike”) remained unfolded for ~20 s (Figure 2D), similar to PpiA WT (Figure 1B), and subsequently folded slower than PpiB WT (Figure 2; compare D to B). In contrast to PpiA, SecB had minimal effect on the fast folding of either the WT PpiB (Figure 2; compare E to B) or the PpiB(rheo) mutant (compare F to C). However, SecB caused a significant delay in the folding of the slow-folding PpiB_>A,6plet_ mutant (compare G to D).

Despite efforts, we were not successful in investigating the effect of a second chaperone, TF, on the folding of our structural twin proteins by global HDX-MS due to technical issues; the presence of TF distorted the protein spectra and prevented reliable data analysis.

In conclusion, our HDX-MS findings seem to suggest that SecB can delay the folding of slow-folding proteins [PpiA WT, PpiA(Δrheo), PpiA(3A), and PpiB_>A,6plet_] but its presence does not seem to affect fast-folding proteins [PpiB WT and PpiB(rheo)].

### 2.2. TF and SecB Have Differential Effects on Protein Translocation In Vitro

To investigate whether the effect SecB had on folding also impacts protein translocation efficiency, we employed an established in vitro translocation assay [13], using a Prl translocase that, unlike the wild-type SecYEG translocase, allows some secretion of proteins in the absence of signal peptides [5,31,32]. The proteins of interest were fused C-terminally to WT PhoA (designated as PpiX_PhoA_) allowing for reliable detection of the hybrid protein using highly specific antibodies. We have previously used similar fusion constructs to assess in vivo secretion as a functional readout of folding behavior [28,29].

**Figure 3:**
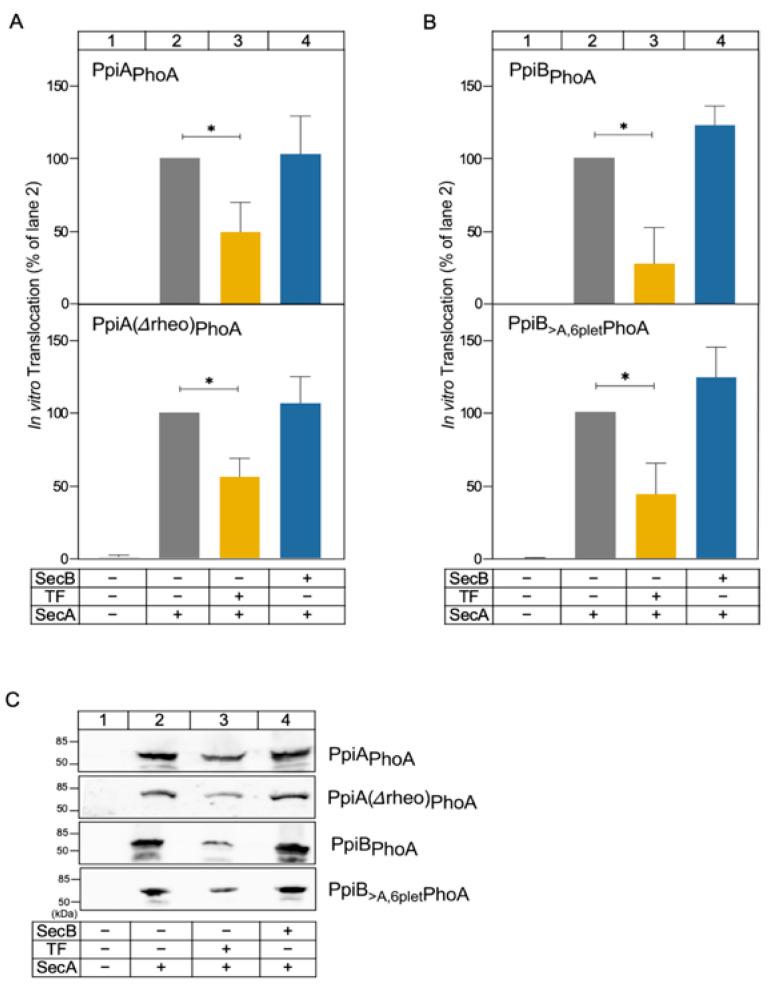
Effect of TF and SecB on the in vitro translocation of PpiX_PhoA_ substrates. (**A**,**B**) Quantification of the in vitro translocation of the indicated proteins (5 µM unfolded PpiX_PhoA_ clients, 0.4 µM SecA, 0.4 µM SecY_PrlA4_EG-IMVs, 1 mM ATP; 10 min, 37 °C) in the presence or absence of TF or SecB (as indicated; (**A**) PpiA_PhoA_ and its derivative; (**B**) PpiB_PhoA_ and its derivative). Following proteinase K treatment (1 mg/mL, 20 min on ice), TCA precipitation, SDS-PAGE, and immunostaining with anti-PpiA/B antibodies, signals from translocated proteins were quantified. Translocation efficiency in the absence of chaperones (i.e., lane 2) was set to 100%, and all others were expressed relative to this value. Mean values ± SD are shown; *n* = 3. Statistical significance was calculated using one-sample *t*-tests (95% confidence interval). * = *p* < 0.05. (**C**) Representative western blots from the experiments described in (**A**,**B**).

Inverted membrane vesicles (IMVs), prepared from cells overexpressing SecY_PrlA4_EG (hereafter SecY_PrlA4_EG-IMVs; 0.4 µM), were incubated with 0.4 µM SecA, 5 µM urea-unfolded clients, and 1mM ATP for 10 min at 37 °C, in the absence or presence of either 20 µM TF or 40 µM SecB. Translocation was terminated by transfer of samples on ice and proteinase K treatment. Successfully translocated clients entered the lumen of IMVs and became protected from protease degradation. Following TCA precipitation, SDS-PAGE, and western blot analysis, translocation efficiency was determined by quantifying the amount of proteinase K-resistant client relative to the client input amount. The translocation efficiency in the absence of chaperones was set as 100%; all other values were expressed relative to this value (Figure 3).

As expected, none of the fusion proteins was translocated in the absence of SecA (Figure 3, lane 1). In contrast, in the presence of SecA (without chaperones), both WT PpiA (Figure 3A; top) and PpiB (Figure 3B; top), as well as representative mutants (bottom), were efficiently translocated into the lumen of IMVs (Figure 3, lane 2). Although our client proteins spanned a range of folding behaviours (shown in Figure 1 and Figure 2), addition of TF significantly inhibited the translocation efficiency of all clients (Figure 3; compare lane 3 to lane 2), while SecB had no significant effect (compare lane 4 to lane 2).

In summary, our results show that, unlike SecB, TF inhibits the Sec-dependent translocation of the PpiA/B and derivative proteins under the same conditions.

### 2.3. SecB Is Dominant over TF in Protein Translocation In Vitro

**Figure 4:**
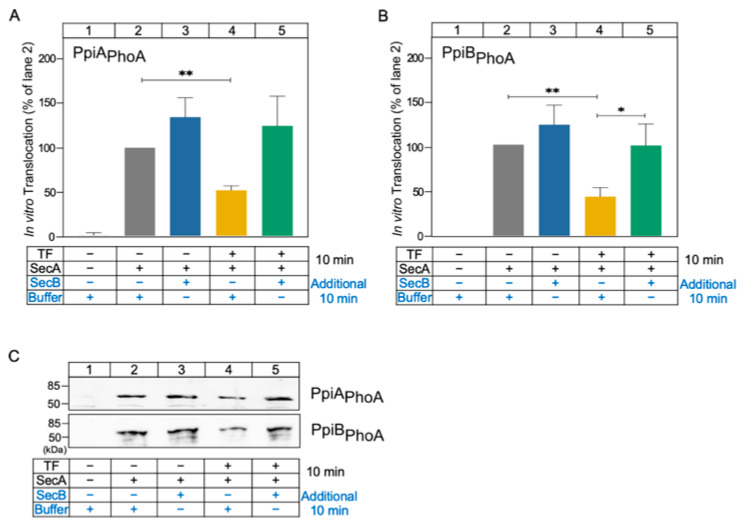
SecB restores translocation of TF-bound substrates in vitro. (**A**,**B**) Quantification of the in vitro translocation of the indicated PpiX_PhoA_ clients (as in Figure 3), following a two-step assay. During the first phase, PpiA_PhoA_ (**A**) and PpiB_PhoA_ (**B**) clients were incubated for 10 min at 37 °C (conditions in black). Then, SecB or buffer was added and incubation continued for an additional 10 min at 37 °C (as indicated, conditions in blue). Following proteinase K treatment, TCA precipitation, SDS-PAGE, immunostaining with anti-PpiA/B antibodies and quantification, translocation of clients was determined, as in Figure 3. Mean values ± SD are shown; *n* = 3. Statistical significance was determined by one-sample t test or unpaired two-tailed *t*-test: * = *p* < 0.05; ** = *p* < 0.01. (**C**) Representative western blots from the experiments described in (**A**,**B**).

Given that TF and SecB might have different roles in protein translocation, we tested whether the inhibitory effect of TF could be overcome by SecB. To study this, we developed a two-phase in vitro translocation assay to mimic the potential client handover from TF to SecB, using the two WT proteins PpiA_PhoA_ and PpiB_PhoA_. During the first phase, clients were incubated in the absence or presence of TF for 10 min (as in Figure 3; Figure 4, conditions indicated in black). Then, SecB or buffer was added, and incubation continued for an additional 10 min (Figure 4, conditions indicated in blue). Signals from translocated proteins were quantified (as in Figure 3). For each client, translocation efficiency in the absence of chaperones was considered 100%; all other values were expressed relative to this.

When added alone, TF significantly reduced the translocation efficiency of both substrates (Figure 4; compare lane 4 to lane 2; panel A, *p* = 0.0036 for PpiA_PhoA_ and panel B, *p* = 0.0097 for PpiB_PhoA_), while SecB had no significant effect (Figure 4; compare lane 3 to lane 2; panel A, *p* = 0.1140 for PpiA_PhoA_ and panel B, *p* = 0.2240 for PpiB_PhoA_). Both results as well as the controls (lanes 1 and 2) were in line with the effects shown in Figure 3. Strikingly, following addition of SecB on samples that have been pre-incubated with TF, translocation was restored to control levels for either client (Figure 4; *p* = 0.0611 for PpiA_PhoA_ and *p* = 0.0367 for PpiB_PhoA_ for comparison of lane 5 with lane 4, and *p* = 0.3283 for PpiA_PhoA_ and *p* = 0.9645 for PpiB_PhoA_ for comparison of lane 5 with lane 2). The comparison between lanes 5 and 3 demonstrates that once SecB is present it overrides any TF effect (Figure 4; *p* = 0.6997 for PpiA_PhoA_ and *p* = 0.2875 for PpiB_PhoA_ for comparison of lane 5 with lane 3).

These results suggested that the SecB effect is dominant over the TF effect. TF-bound substrates either remain in a translocation-competent state but cannot be delivered to the translocase unless they are taken over by SecB, or get trapped in a translocation-incompetent state that can be reversed by SecB; hence, they can re-enter the Sec pathway.

### 2.4. SecB Improves proPpiA Solubility but Does Not Enhance Its In Vitro Translocation

The presence of a signal peptide (SP) is known to affect preprotein folding, solubility and chaperone interactions [29,33]. To probe into this, we compared the solubility of proPpiA with its mature counterpart PpiA and tested whether SecB and TF chaperones had an effect on the solubility of the preprotein.

PpiA and proPpiA urea-purified proteins were allowed to refold by dilution in aqueous buffer (18 µM protein; 0.2 M urea; 5 mM DTT; 1 mM EDTA; 30 min, 37 °C). To compare the amount of protein that remained soluble under these conditions, samples were centrifuged and the aggregated material was removed. Samples, before and after centrifugation (20,000× *g*, 4 °C, 10 min), were analyzed on SDS-PAGE (Figure S1) and quantified using ImageJ. The amount of protein before centrifugation was considered 100%; all other values were expressed relative to this value.

**Figure 5:**
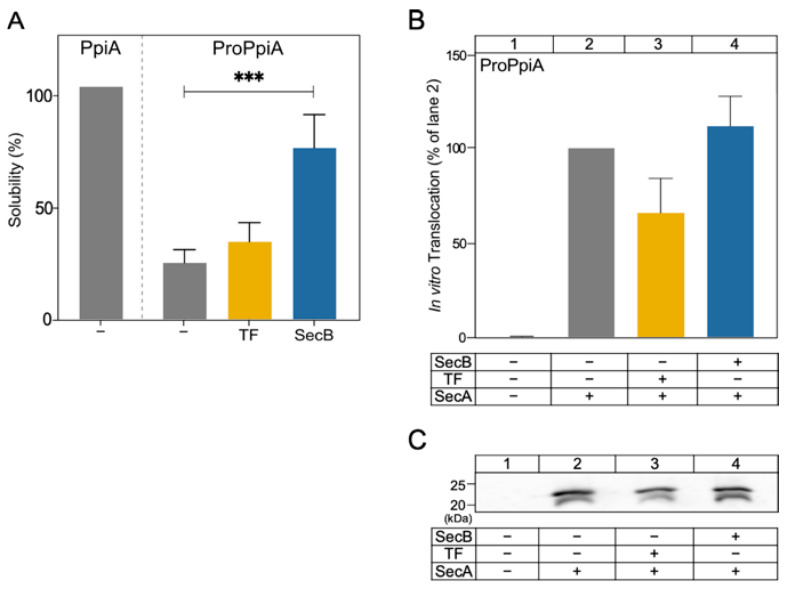
Chaperone effects on the solubility and the in vitro translocation of proPpiA. (**A**) Urea-purified PpiA or proPpiA (as indicated) were diluted in aqueous buffer in the absence or presence of TF or SecB, and incubated for 30 min at 37 °C. Aggregated (insoluble) fraction was separated by centrifugation (20,000× *g*, 4 °C, 10 min). Protein samples before and after centrifugation were analyzed by SDS-PAGE, Coomassie blue-stained, and quantified by ImageJ. The protein amount before centrifugation was set as 100%; all other values were expressed relative to this value. For PpiA, *n* = 1; for proPpiA *n* ≥ 3. Mean values ± SD are shown. Statistical significance was determined by unpaired two-tailed *t*-test: *** *p* < 0.001. (**B**) Quantification of proPpiA in vitro translocation, as in Figure 3 (0.4 µM SecA, 0.4 µM SecYEG-IMVs, 1 µM proPpiA; 10 min; 37 °C). Following proteinase K treatment (1 mg/mL, 20 min on ice), TCA precipitation, SDS-PAGE and immunostaining using anti-PpiA antibodies, signals from translocated proteins were quantified using ImageJ. Translocation efficiency in the absence of chaperones was set to 100% (lane 2); all others were expressed relative to this value. Mean values ± SD are shown; *n* = 3. Statistical significance was determined by one-sample *t*-tests. (**C**) Representative western blots from the experiments presented in (**B**).

Unlike PpiA, which remains soluble under these conditions (Figure 5A, lane 1; ~100%), proPpiA showed a strong tendency to aggregate (lane 2; ~30% remains soluble). Addition of TF (40 µM) in the dilution buffer did not significantly affect solubility (Figure 5A, lane 3; *p* = 0.1068 for comparison with buffer control), whereas that of SecB (108 µM) significantly improved solubility of proPpiA (lane 4; ~70% remains soluble; *p* = 0.0004 as compared to buffer control of lane 2).

Our results suggest that SecB, unlike TF, can improve the solubility of proPpiA, enabling the preprotein to maintain a translocation-competent state.

To test whether SecB and TF chaperones have an effect on the secretion of proPpiA, an in vitro translocation assay was employed, now using the wild-type SecYEG translocase (0.4 µM SecA, 0.4 µM SecYEG-IMVs, 1 µM proPpiA, 1mM ATP; 10 min, 37 °C) in the absence or presence of chaperones (4 µM TF or 8 µM SecB), as in Figure 3.

Similarly to PpiA_PhoA_ (Figure 3A), TF had an inhibitory effect on the translocation of proPpiA, although not reaching statistical significance (Figure 5B; compare lane 3 to lane 2; *p* = 0.0821), while SecB did not cause a change in the translocation of proPpiA (Figure 5B; compare lane 4 to lane 2; *p* = 0.3253).

### 2.5. SecB and TF Effects on Preprotein Translocation Seem to Be Client Specific

To examine whether the effects of TF and SecB are universal on multiple clients, we selected three additional preproteins (i.e., proMBP, proOmpA, and proPhoA) and studied their in vitro translocation in the absence or presence of chaperones, as in Figure 5B.

**Figure 6:**
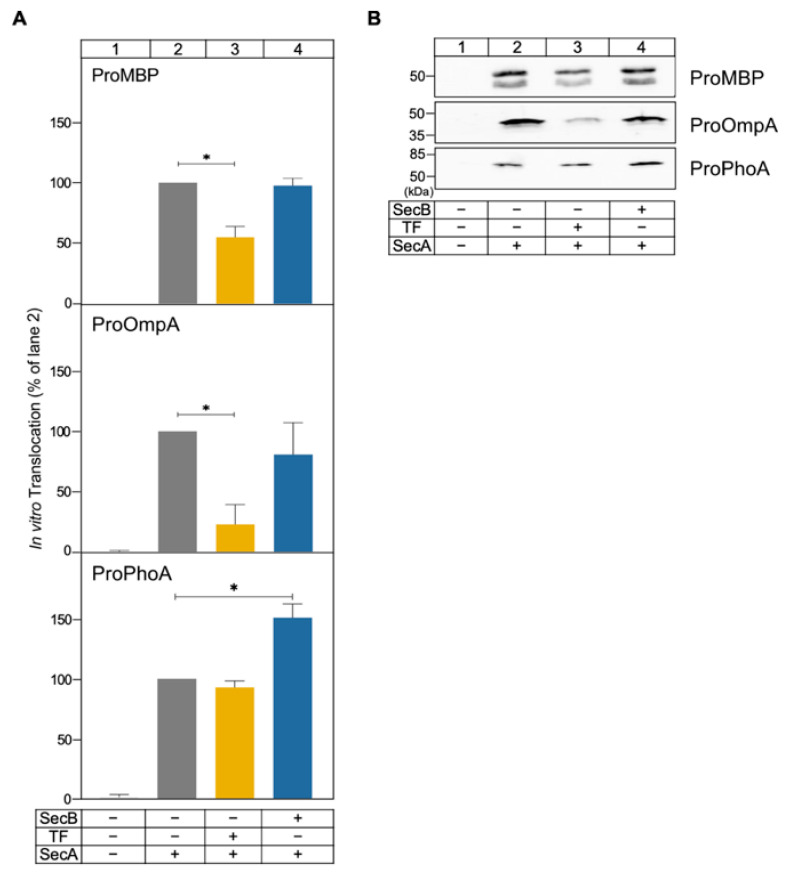
Effects of molecular chaperones on in vitro translocation of model preproteins. (**A**) Quantification of the in vitro translocation of the indicated preproteins, as in Figure 3 (0.4 µM SecA; 0.4 µM SecYEG-IMVs; 3 µM clients; 1 mM ATP; 10 min; 37 °C). Following proteinase K treatment (1 mg/mL, 20 min on ice), TCA precipitation, SDS-PAGE and immunostaining using substrate-specific antibodies, signals from translocated proteins were quantified using ImageJ. Translocation efficiency in the absence of chaperones was set to 100% (lane 2); all others were expressed relative to this value. Mean values ± SD are shown; *n* = 3. Statistical significance was determined by one-sample *t*-tests: * = *p* < 0.05. (**B**) Representative western blots from experiments presented in (**A**).

We observed that TF inhibited translocation of both proMBP and proOmpA, yet to a differential degree (Figure 6; compare lane 3 to 2; *p* = 0.0122 for proMBP and *p* = 0.0145 for proOmpA), while SecB had no measurable impact on either client (Figure 6; compare lane 4 to 2; *p* = 0.5564 for proMBP and *p* = 0.3361 for proOmpA). For proPhoA SecB significantly enhanced translocation (Figure 6; compare lane 4 to 2; *p* = 0.0159), whereas TF had no detectable effect (compare lane 3 to 2; *p* = 0.1448).

Our findings indicate that the combined properties of SPs and MDs dictate chaperone specificity and translocation efficiency, with both TF and SecB acting in a substrate-specific manner.

## 3. Discussion

Despite extensive studies on the SecA-dependent secretion pathway, the contribution of molecular chaperones to the folding trajectory and secretion of preproteins remains unclear [13,34,35]. Using a secreted (PpiA) and a cytoplasmic (PpiB) structural twin pair, we have previously shown that signal peptides and mature domain features allow preproteins to delay their folding during their cytoplasmic transit [28,29]. Here, we used the same pair to test whether two known secretory chaperones, SecB and trigger factor (TF) [11,13,36,37,38], can impact folding of exported proteins and/or SecA-dependent secretion. Although TF is known to bind co-translationally to nascent chains, here we evaluate only post-translational effects.

Global HDX-MS analysis revealed that SecB drastically delays the folding of secreted PpiA, and all derivatives [PpiA(Δrheo) and PpiA(3A)] that exhibit prolonged unfolded states (Figure 1). Cytoplasmic proteins that fold faster, such as PpiB and its derivative PpiB(rheo), are not affected by SecB (Figure 2B,C,E,F). The only exception to this rule is a PpiB derivative (PpiB_>A,6plet_) whose folding became delayed due to mutations in its mature domain that resulted in a more PpiA “look-alike” structure. Hence, it resembled more the folding of PpiA and less that of PpiB (compare Figure 2D to Figure 1B and Figure 2B) [28]. SecB delayed folding of PpiB_>A,6plet_ as well (Figure 2D,G). Our observations support a model in which SecB recognizes and binds clients with delayed/weakened initial folding steps and extends their time-window of translocation-competent conformations. If this is a crucial step to optimize handover to SecA [26,39,40], it would be anticipated that the presence of SecB would enhance the secretion efficiency of those clients. Instead, we see that the addition of SecB did not significantly alter the translocation efficiency of these clients in vitro (Figure 3). Two explanations come to mind: (i) either these substrates are already slow-folders and translocation-efficient, making the additional delay that SecB offers redundant, or (ii) the presence of SecA in the reactions is dominant and makes any SecB contribution negligible.

We were not able to collect global HDX-MS information on the effect of TF on client folding under the same conditions, due to technical reasons. However, we have shown that TF interferes with the in vitro translocation of all PpiA/PpiB clients, fast or slow folders (Figure 3). The ~50% inhibition of the post-translational secretion of these substrates in the presence of TF is in agreement with previous observations using proOmpA, a known TF client [13,41,42]. Interestingly, the addition of SecB in such a reaction relieves the inhibition of TF on PpiA/B secretion (Figure 4), similarly to previous observations with proOmpA [13].

The contribution of the signal peptide in the interaction of secretory proteins with chaperones was tested by using proPpiA. The signal peptide increased the aggregation propensity of PpiA (Figure 5A), in line with what has been seen for MBP [8]. Only SecB can rescue proPpiA from aggregation; the effect of TF in the solubility of proPpiA is negligible. This contrasts what was previously seen using an aggregation-prone proOmpA derivative, where both TF and SecB significantly increased its solubility [13]. Still, TF interferes with the secretion of proPpiA, while the effect of SecB on in vitro translocation of proPpiA seems negligible (Figure 5B), similar to the effects observed with PpiA (Figure 3). To see whether these effects are universal between preproteins or are substrate specific, three additional preproteins were used: proOmpA, proMBP, and proPhoA. TF demonstrated the maximum inhibition in the secretion of proOmpA (~80% inhibition; Figure 6A) in agreement with previous results [13], exerted a comparable inhibitory effect on the secretion of proPpiA and proMBP, but had no effect in the secretion of proPhoA. On the other hand, SecB had no effect on the translocation of proOmpA and proMBP (Figure 6), similarly to proPpiA (Figure 5B), but improved translocation of proPhoA (Figure 6). Such diversity suggests that the chaperones effects on preprotein secretion are substrate-specific and not universal, likely shaped by the combination of features present on signal peptides and mature domains that are distinctly unique for each preprotein.

The features of the various proteins tested in this study differ considerably. Differences in length and in the number of charged, polar and hydrophobic residues, create unique combinations among the signal peptides alone; e.g., proPpiA has 1 positive charge and 19 hydrophobic residues that fall in two stretches (of 5 to 10 residues); MBP has 3 positive charges close to each other and 17 hydrophobic residues that are spread out (with 4 residues as the longer hydrophobic patch). When these are combined with mature domain features that vary in charged, polar and hydrophobic content, in disulfide bond formation, length and number of hydrophobic patches and disorder, truly unique combinations are created. This variability influences not only their folding rate (e.g., OmpA folds slowly, MBP rapidly, PhoA slowly and depends on disulfide bond formation; and preproteins with signal peptides fold slower than their mature domains) [8,29,38,43], but also their interactions with chaperones [1,35]. It is anticipated that intrinsic differences that affect folding can shape exposure and lifetime of chaperone-binding regions, providing a plausible explanation for the substrate-specific effects observed here with SecB and TF.

In summary, the observations made in this study solidify and expand previous observations concerning the role and interplay of TF and SecB in SecA-dependent preprotein secretion. SecB seems to recognize weak folding proteins and maintain them as translocation-competent for longer (PpiA/B; Figure 1 and Figure 2), perhaps by reverting folding of early foldons as was recently shown for MBP [38]. SecB can also rescue aggregation-prone preproteins such as proPpiA (Figure 5A) and proOmpA [13]. In addition, SecB can improve the translocation efficiency of proPhoA (Figure 6) but not of other clients. TF, on the other hand, interfered with secretion of proPpiA, proOmpA, proMBP, mature PpiA, cytoplasmic PpiB, and derivatives, but not with the secretion of proPhoA (Figure 3, Figure 4, Figure 5 and Figure 6) [13]. What is worth noting is the interplay between SecB and TF observed in our in vitro experiments: in all cases where TF interfered with the secretion of a client, the presence of SecB alleviated this effect, demonstrating the dominance of SecB over TF (this study; [13]). While these are all observations from in vitro assays that isolate co-translational effects of the chaperones on clients, allowing the study of post-translational effects alone, it is already known that the ratio of SecB to TF needs to be optimally retained in vivo, as each one of them can become conditionally toxic in the absence of the other [44,45]. It is evident that the cellular context introduces additional layers of complexity. Client folding, chaperone–client interaction, and translocation are subjected to concomitant effects by more (co-)chaperones and/or the crowded intracellular environment. Future in vivo experiments may reveal relevant client sorting behaviours not evident in reconstituted systems.

## 4. Materials and Methods

### 4.1. Materials/Reagents

The list of buffers (Table S1), strains (Table S2), vectors (Table S3), genes (Table S4), primers (Table S5), and antibodies (Table S6) used in this study can be found in Supplementary Materials. For HDX-MS analysis, D_2_O (Sigma Aldrich, St. Louis, MO, USA, P/N 151882), Urea-d_4_ (Sigma Aldrich, P/N 176087) and Formic Acid (FA, MS-grade, Sigma Aldrich, F0507) were used. Other solvents and chemicals were from Merck (Darmstadt, Germany), Sigma Aldrich or Carl Roth (Karlsruhe, Germany). Proteins were dialyzed using membranes (Medicell Membranes Ltd., London, UK) and concentrated using centrifugal concentrators (VivaSpin, Littleton, MA, USA).

### 4.2. Molecular Cloning

Restriction sites and mutations on genes (Table S4) were added by PCR using PFU Ultra Polymerase (Stratagene, La Jolla, CA, USA), primers (Table S5), and vectors (Table S3) [46].

### 4.3. Bacterial Growth for Induced Protein Expression

Plasmids were transformed into *E. coli* cells; Lemo21(DE3) was used for proMBP; BL21.19(DE3) [47] for SecB, SecA, and TF; BL31(DE3) for SecYEG-IMVs; Tuner (DE3) for PpiA/B derivatives; MC4100 for PhoA fusions [48,49] (Table S2). Bacterial cells were grown in LB medium, at 37 °C, until OD_600_ = 0.6 induced with 0.1–1 mM IPTG or 1 mM arabinose, for 3 h, at 37 °C and harvested by centrifugation (5000× *g*, 4 °C, 15 min). Cell pellets were stored at −20 °C until purification.

### 4.4. Purification of His-Tagged Proteins

His-tagged cytoplasmic proteins and preprotein mature domains were purified under native conditions, whereas preproteins and PhoA-fusion proteins were purified under denaturing conditions, using Ni-NTA (Qiagen, Hilden, Germany) and following the manufacturer instructions. Briefly, cell pellets were resuspended in Buffer A (native) or Buffer E (denaturing) and lysed using a French press. Lysates were centrifuged (15,000 rpm, 4 °C, 30 min), and the supernatant containing soluble proteins was collected directly (soluble purification), while the insoluble pellet was solubilized in 8M urea (Buffer F; o/n, 4 °C) for denatured purification. Both preparations were applied to Ni^+2^-NTA resin (Qiagen) and washed sequentially with Buffer A/B (native) or Buffer G/H (denaturing). Proteins were eluted with 100 mM Imidazole (Buffer C or Buffer H, respectively) and stored in Buffer D or I, respectively, at −20 °C.

### 4.5. Purification of SecB

Untagged SecB was purified as previously described [13,38,50], using ion exchange chromatography followed by size exclusion chromatography (SEC). Briefly, cells were lysed by French Press in buffer S. The soluble fraction was applied to a Q-Sepharose column and then NaCl was diluted with buffer K. The diluted supernatant was loaded onto a Q FF column and eluted with a NaCl (200–400 mM) gradient, by adding buffer U, followed by SEC on a Superdex S200 26/600 (GE HealthCare, Chicago, IL, USA) column under 50 mM NaCl (buffer M). Purified SecB was dialyzed into buffer N for long-term storage.

### 4.6. Purification of SecA

Untagged SecA was purified as previously described [13,38,51], by Cibacron affinity chromatography followed by SEC. Briefly, cells were lysed by French Press in Buffer F, followed by centrifugation. Supernatant was eluted from Cibacron column using a linear gradient of KCl (0.2 to 1.2 M) by introducing buffer P with increased ionic strength. Fractions containing SecA (observed on SDS-PAGE) were pooled, concentrated, and further purified by SEC on a Superdex S200 26/600 column with high-salt (buffer P) and then low-salt (buffer M). Purified SecA was dialyzed into buffer N and stored at −20 °C.

### 4.7. Purity and Concentration Check

Protein purity was examined using SDS-PAGE and Coomassie staining. Protein concentrations were determined using a Nanodrop spectrophotometer (Thermo Scientific, Waltham, MA, USA) based on the molecular weight and extinction coefficient of each protein, calculated with the ExPASy ProtParam tool (http://web.expasy.org/protparam/).

### 4.8. IMVs Purification

IMVs were purified as previously described [13]. Briefly, harvested cells were resuspended in Buffer S and lysed by French press at ~1200 psi. The membrane-containing pellet was resuspended in Buffer S after high-speed centrifugation at 32,000 rpm and homogenized using a Dounce homogenizer. Membranes were further purified by sucrose gradient (1.1 to 1.9 M in buffer A) ultracentrifugation. IMVs were collected at the third layer (1.5 M sucrose) and treated with buffer T (with extra 6 M urea). The samples were subjected to sucrose exchange with buffer U, then washed and resuspended in Buffer T and stored at −80 °C in small aliquots.

### 4.9. In Vitro Translocation

In vitro translocation reactions were assembled by mixing 0.4 µM SecA, 0.4 µM SecYEG or SecY_PrlA4_EG-IMVs, 5 µM mature domain proteins or 1 µM proPpiA or 3 µM other proproteins, 1 mM ATP, −/+ chaperones (TF_2_ and/or SecB_4_; in double the amount of the clients). Reactions were incubated at 37 °C for 10 min. Translocation into the lumen of the IMVs was determined as protection from subsequent proteinase K treatment (1 mg/mL; 20 min, 4 °C). Following TCA precipitation, proteins were analyzed by SDS–PAGE, transferred onto nitrocellulose, immunostained with specific antibodies (Table S6), and visualized following incubation with SuperSignal West Pico Plus chemiluminescence substrate using LAS4000 (GE HealthCare, Chicago, IL, USA). Prestained protein markers that were used as controls in the same gels were visualized using LAS4000 in digitization mode. The two gel images were aligned and overlapped. Signals from translocated proteins were quantified using ImageJ (https://imagej.net/ij/index.html) and expressed relative to the signal of a translocation reaction with SecA only (no chaperone), which is set as 100%; all other values were expressed relative to this value.

### 4.10. Solubility Assays

proPpiA dialyzed in buffer H was pre-treated as described [29]. The refolding experiment was initiated by diluting the denatured protein in aqueous buffer (final 0.2 M urea; 5 mM DTT; 1 mM EDTA; 18 µM protein). Following dilution, an aliquot of the sample was kept on ice as a control for total protein content. Samples were incubated for 30 min at 30 °C and then centrifuged (20,000× *g*, 4 °C, 10 min) to remove aggregates. The supernatant contained only soluble proteins. At total of 4 µg protein of the total protein content sample and the same volume of supernatant were analyzed on SDS-PAGE and Coomassie-blue stained. Protein quantification was determined using ImageJ on those gels. The soluble proteins were expressed as % of the total protein content (set as 100%).

### 4.11. Global Hydrogen-Deuterium Exchange (HDX) Mass Spectrometry (MS) Experiments

HDX buffers and conditions have been described in detail [29]. Briefly, proteins were dialyzed in buffer Q, treated for 40 min at 37 °C and reduced (100 mM DTT; ice) for complete denaturation. Refolding was initiated by diluting the pre-treated denatured protein in aqueous buffer (final concentrations 0.2 M urea; 18 µM protein), with or without 108 µM SecB, at 25 °C. At various folding time points (10 s, 20 s, 40 s, 60 s, 2.5 min, 5 min, 15 min, 30 min, and 1 h, as indicated), protein samples were pulse labelled in D_2_O [pD 8.0; 95.52% (*v*/*v*)] for 100 s. Natively folded protein controls from soluble protein purification, whenever applicable, were used as controls (pulse labelled in D_2_O for 100 s). Denatured controls were pulse labelled in 6 M Urea-d_4_ (pD 8.0; 95.52% (*v*/*v*) D_2_O), 5 mM DTT, 1 mM EDTA on ice, for 100 s (t0 control) and 1 h (FD control). Labelling was quenched with prechilled formic acid (to pD 2.5), snap-frozen in liquid nitrogen, and stored at −80 °C until MS analysis. Conditions for MS analysis and determination of D uptake were as described [29]. A single charged state of highest intensity was selected in order to monitor the folding states during the indicated time course. A continuous folding colour map from discrete time points was created by following a previously published procedure [29].

### 4.12. Statistical Analysis

Statistical analysis of replicates was performed using Excel, GraphPad Prism (Version 10.6.1, GraphPad Software, LLC, Boston, MA, USA), and Python (Version 3.6.3, The Python Software Foundation, Wilmington, DE, USA). Error bars represent standard error or standard deviation, as indicated.

## Data Availability

Plasmids and other materials generated in this study are available upon request from the lead contact, Kurt Vermeire (kurt.vermeire@kuleuven.be).

## Supplementary Materials

The supporting information can be downloaded at https://www.mdpi.com/article/10.3390/ijms262311485/s1. References [4,5,8,28,29,44,46,47,48,49,50,51] are cited in the supplementary materials.

## Abbreviations

The following abbreviations are used in this manuscript:

*E. coli*	*Escherichia coli*
D	Deuterium
FA	Formic acid
HDX-MS	Hydrogen deuterium exchange mass spectrometry
IMVs	Inverted membrane vesicles
MBP	Maltose binding protein
MD	Mature domain
MTSs	Mature domain targeting signals
OmpA	Outer membrane protein A
PhoA	Alkaline phosphatase
PpiA	Peptidyl prolyl cis trans isomerase A
PpiB	Peptidyl prolyl cis trans isomerase B
Prl	Protein localization
SEC	Size exclusion chromatography
SP	Signal peptide
TF	Trigger factor
WT	Wild type
