# Supplementary material for: Effect of the Bacterial Chaperones SecB and Trigger Factor (TF) on the Folding Dynamics and In Vitro Translocation of Cytoplasmic and Secretory Model Proteins
Source: Int J Mol Sci. 2025 Nov 27;26(23):11485. doi: 10.3390/ijms262311485 (PMC12691745; doi:10.3390/ijms262311485)
Supplement: Supplementary file 1 [file ijms-26-11485-s001.zip › ijms-3983837-supplementary.pdf]

Supplemental Information

Effect of the Bacterial Chaperones SecB and Trigger Factor (TF) on the Folding Dynamics and In Vitro Translocation of Cytoplasmic and Secretory Model Proteins

Table of contents

Supplemental figures:

Figure S1: SDS-PAGE of in vitro solubility analysis of (pro)PpiA in the presence or absence of chaperones

Supplemental tables:

Table S1: Buffers
Table S2: Strains
Table S3: Vectors
Table S4: Cloned genes
Table S5: Primers
Table S6: Antibodies

Supplemental figure

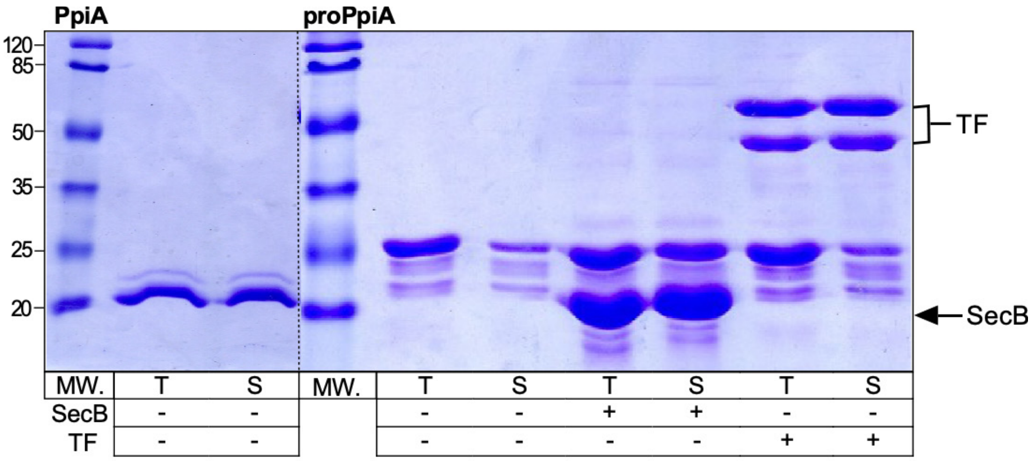

Figure S1. SDS-PAGE of in vitro solubility analysis of (pro)PpiA in the presence or absence of chaperones. Purified proteins were incubated with or without TF or SecB for 30 min at 30 °C, and solubility was assessed by centrifugation followed by SDS-PAGE. Samples were separated into total (T) and soluble (S) fractions. The resulting bands were visualized by Coomassie staining. The proportion of soluble protein relative to total protein was quantified and is presented in Figure 5.

Supplemental Tables:

Table S1: *Buffers*

Buffer A	50 mM Tris-HCl pH 8, 1 M NaCl, 5mM Imidazole, 5% glycerol
Buffer B	50 mM Tris-HCl pH 8, 50 mM NaCl, 5mM Imidazole, 5% glycerol
Buffer C	50 mM Tris-HCl pH 8, 50 mM NaCl, 100mM Imidazole, 5% glycerol
Buffer D	50 mM Tris-HCl pH 8, 50 mM NaCl, 50% glycerol
Buffer E	50 mM Tris-HCl pH 8, 500 mM NaCl, 5mM $MgCl_2$, 5mM Imidazole, 5% glycerol
Buffer F	50 mM Tris-HCl pH 8, 500 mM NaCl, 5mM $MgCl_2$, 5mM Imidazole, 5% glycerol, 8M Urea
Buffer G	50 mM Tris-HCl pH 8, 500 mM NaCl, 5mM $MgCl_2$, 5mM Imidazole, 5% glycerol, 6M Urea
Buffer H	50 mM Tris-HCl pH 8, 50 mM NaCl, 5mM $MgCl_2$, 5mM Imidazole, 5% glycerol, 6M Urea
Buffer I	50 mM Tris-HCl pH 8, 50 mM NaCl, 2.5mM $MgCl_2$, 10% glycerol, 6M Urea
Buffer J	50 mM Tris-HCl pH 7.4, 400 mM NaCl, 1mM $MgCl_2$
Buffer K	50 mM Tris-HCl pH 7.4
Buffer L	50 mM Tris-HCl pH 7.4, 200 mM NaCl
Buffer M	50 mM Tris-HCl pH 8.0, 50 mM NaCl
Buffer N	50 mM Tris-HCl pH 8.0, 50 mM NaCl, 50% glycerol
Buffer O	50 mM Tris-HCl pH 7.4, 200 mM KCl, 1mM $MgCl_2$, 10% glycerol
Buffer P	50 mM Tris-HCl pH 7.4, 1.2 M KCl, 10% glycerol
Buffer Q	25 mM Tris-HCl pH 8.0, 25 mM KCl, 8M urea
Buffer R	25 mM Tris-HCl pH 8.0, 25 mM KCl
Buffer S	50 mM Tris-HCl pH 8.0, 20% glycerol
Buffer T	50 mM Tris-HCl pH 8.0, 50 mM KCl, 5mM $MgCl_2$
Buffer U	0.2 M sucrose, 50 mM Tris pH 8.0, 50 mM KCl

Table S2: *Strains*

***E. coli* strain**	**Description**	**Reference/source**	**Used for purification**
Lemo21(DE3)	T7 RNA polymerase gene under the control of the lacUV5 promoter.	New England BioLabs	proMBP
BL21.19(DE3)	secA13 (Am) supF (Ts) trp (Am) zch::Tn10 recA::cat clpA::kan)	[1]	SecB, SecA, TF
BL31 (DE3)	Non ts, spontaneous revertant of BL21.19(DE3).	[2]	SecYEG-IMVs, SecYprlA4EG-IMVs
Tuner (DE3)	lacZY deletion mutant of BL21	Novagen	PpiA/B and derivatives, proPpiA, proOmpA, proPhoA
MC4100	F-araD139 ¢(argF-lac)U169 rpsL150 (StrR) relA1 flbB5301 deoC1 pstF25 rbsR	[3-5]	PhoA fused proteins

Table S3: *Vectors*

Vector	Antibiotic resistance	promoter	Origin of replication	Reference/Source
pET22b	Ampicillin	T7(lac)	pBR322	Novagen (https://www.merckmillipore.com/)
pBAD501	Gentamycin	ara	p15A/	[6]

Table S4: *Cloned genes*

Gene	Uniprot accession number	Plasmid name	Vector	Description/reference
proPpiA-His	P0AFL3	pIMBB1042	pET22b	[7]
ppiA-His	P0AFL3	pIMBB1043	pET22b	[7]
ppiB-His	P23869	pIMBB1085	pET22b	[8]
ppiB>A,6plet-His		pLMB2087	pET22b	[8]
ppiB(rheo)-His		pLMB2090	pET22b	[9]
ppiAΔrheo-His		pLMB2089	pET22b	[9]
ppiA(3A)-His		pLMB2195	pET22b	[9]
$ppiA_{PhoA}$-His		pIMBB1571	pBAD501	[8]
ppiBPhoA-His		pIMBB1584	pBAD501	[8]
ppiB>A,6pletPhoA-His		pLMB2209	pBAD501	[8]
ppiA(Δrheo)PhoA-His		pLMB2259	pBAD501	PpiA(*Δrheo)* was amplified from pLMB2089 [9] using primers X2469 and X1282 and inserted pBAD501 [6] after NdeI-HindIII digestion.
proPhoA-His	P00634	pIMBB882	pET22b	[10]
proOmpA-His	P0A910	pLMB1835	pET22b	[10]
proMBP-His	P0AEX9	pIMBB1147	pET22b	[10]
secB	P0AG86	pIMBB351	pJW25	[11]
His-*tig*	P0A850)	pCold-TF	pCold	Takara (https://www.takarabio.com)
secA	P10408	pIMBB1280	pET3a	[12]
His-*secY*(prlA4)-*EG*		pIMBB842	pET610	[8]

Table S5*: Primers*

Primer	Forward/ Reverse	Gene	Restriction site or/and mutation inserted	Sequence (5'-3')
X1282	R	*ppiA*	HindIII	5′ CCC AAG CTT CGG CAG GAC TTT AGC GGA AAG GAT AA 3′
X2469	F	*PpiA(Δrheo)*	NdeI	5′ GGGAATTC CAT ATG GTA TTG TTG ACA ACC TCA 3′

Table S6*: Antibodies*

Antibodies	Animal source	Reference
a-PpiA	Rabbit[a]	This study
a-PpiB	Rabbit	This study
a-PhoA	Rabbit	[7]
a-His	Mouse	SEROTEC
α-Rabbit	Goat	Jackson ImmunoResearch Europe Ltd
α-Mouse	Goat	Jackson ImmunoResearch Europe Ltd

[a]Rabbit polyclonal antibodies against the indicated purified proteins were raised by Davids Biotechnologie, Germany.

References

1. Mitchell, C.; Oliver, D. Two distinct ATP-binding domains are needed to promote protein export by Escherichia coli SecA ATPase. *Mol Microbiol* **1993,** *10*, (3), 483-97.
2. Chatzi, K. E.; Sardis, M. F.; Tsirigotaki, A.; Koukaki, M.; Šoštarić, N.; Konijnenberg, A.; Sobott, F.; Kalodimos, C. G.; Karamanou, S.; Economou, A. Preprotein mature domains contain translocase targeting signals that are essential for secretion. *J. Cell Biol.* **2017,** *216*, (5), 1357-1369.
3. Casadaban, M. J. Transposition and fusion of the lac genes to selected promoters in Escherichia coli using bacteriophage lambda and Mu. *J Mol Biol* **1976,** *104*, (3), 541-55.
4. Genevaux, P.; Schwager, F.; Georgopoulos, C.; Kelley, W. L. Scanning mutagenesis identifies amino acid residues essential for the in vivo activity of the Escherichia coli DnaJ (Hsp40) J-domain. *Genetics* **2002,** *162*, (3), 1045-53.
5. Ullers, R. S.; Ang, D.; Schwager, F.; Georgopoulos, C.; Genevaux, P. Trigger Factor can antagonize both SecB and DnaK/DnaJ chaperone functions in *Escherichia coli*. *Proc. Natl. Acad. Sci. U. S. A.* **2007,** *104*, (9), 3101-6.
6. Guzman, L. M.; Belin, D.; Carson, M. J.; Beckwith, J. Tight regulation, modulation, and high-level expression by vectors containing the arabinose PBAD promoter. *J. Bacteriol.* **1995,** *177*, (14), 4121-30.
7. Gouridis, G.; Karamanou, S.; Gelis, I.; Kalodimos, C. G.; Economou, A. Signal peptides are allosteric activators of the protein translocase. *Nature* **2009,** *462*, (7271), 363-7.
8. Smets, D.; Tsirigotaki, A.; Smit, J. H.; Krishnamurthy, S.; Portaliou, A. G.; Vorobieva, A.; Vranken, W.; Karamanou, S.; Economou, A. Evolutionary adaptation of the folding pathway for secretability. *(submitted)* **2022.**

9. Smets, D.; Smit, J.; Xu, Y.; Karamanou, S.; Economou, A. Signal peptide-rheostat dynamics delay secretory preprotein folding. *J. Mol. Biol.* **2022,** *434*, (19), 167790.
10. Tsirigotaki, A.; Chatzi, K. E.; Koukaki, M.; De Geyter, J.; Portaliou, A. G.; Orfanoudaki, G.; Sardis, M. F.; Trelle, M. B.; Jorgensen, T. J. D.; Karamanou, S., et al. Long-Lived Folding Intermediates Predominate the Targeting-Competent Secretome. *Structure* **2018**.
11. Weiss, J. B.; Ray, P. H.; Bassford, P. J., Jr. Purified secB protein of Escherichia coli retards folding and promotes membrane translocation of the maltose-binding protein in vitro. *Proc Natl Acad Sci U S A* **1988,** *85*, (23), 8978-82.
12. Karamanou, S.; Gouridis, G.; Papanikou, E.; Sianidis, G.; Gelis, I.; Keramisanou, D.; Vrontou, E.; Kalodimos, C. G.; Economou, A. Preprotein-controlled catalysis in the helicase motor of SecA. *EMBO J.* **2007,** *26*, (12), 2904-14.
